# Promoting Physical Activity through Priming the Content of Motivation

**DOI:** 10.3389/fpsyg.2017.01509

**Published:** 2017-09-05

**Authors:** Tom St Quinton

**Affiliations:** Department of Sport, Health and Nutrition, Leeds Trinity University Leeds, United Kingdom

**Keywords:** non-conscious processes, priming, self-efficacy, behavior change, physical activity

## Abstract

Non-conscious processes are important in influencing the performance of a number of behaviors, such as physical activity. One way that such processes can be influenced is through priming. Despite this, approaches within health psychology have predominantly focused on reflective processes with a number of psychological theories dedicated to identifying the predictors of intention. In doing so, critical beliefs and thoughts are first identified and then altered within interventions. Such work has shown limited effectiveness, however, with a gap apparent between what one intends to do and what subsequently ensues. Although there have been attempts to bridge this gap, such as theoretical integration, recent efforts include priming implicit processes. The aim of this commentary is to demonstrate the potential effectiveness of priming non-conscious processes and to suggest that the content of motivation should also succumb to priming influences. This brief review suggests that priming one of the most influential conscious processes, that of self-efficacy, could demonstrate particular effectiveness in promoting physical activity. Thus, the main purpose of the article is to suggest that the content of implicit processes as well their more traditional conscious counterparts may provide useful intervention targets. To achieve this, the article will first introduce the role of non-conscious processes and behavioral priming. Following this, the more common reflective processes will be outlined as well as attempts at theoretical integration. Finally, the article will identify studies priming non-conscious processes and will then suggest priming self-efficacy.

## Non-Conscious Processes

Although behavior can be perceived to be regulated by forethought and an agentic self (Bandura, 1989), research has demonstrated that non-conscious or implicit processes play a crucial role in human functioning (Bargh, 2006). Non-conscious processes can be regarded as either “preconscious” or “postconscious” (Bargh et al., 2012). Preconscious refers to the non-conscious activation of implicit processes based upon the mere perception of a stimuli or environmental influence. Postconscious concerns the non-conscious enactment of previously developed conscious intentions. For example, implementation intentions (Gollwitzer, 1999) have been shown to increase the likelihood of behavioral performance. In this sense, contextual cues can provide the habitual performance of behavior without the need for conscious deliberation (Gardner et al., 2016). Due to the finite nature of consciousness, such automization fulfills the adaptation process and allows consciousness to remain fully stored. The interactions between the two processes and the ways in which they govern behavior are represented in a variety of ways within a number of dual-process models (e.g., Strack and Deutsch, 2004; Hall and Fong, 2007; Hofmann et al., 2008).

One specific health behavior, that of physical activity, has been found to be influenced by implicit attitudes. For example, those who exercise more have greater positive implicit attitudes toward exercise and physical activity (Markland et al., 2015). Similarly, Antoniewicz and Brand (2016) found automatic evaluations to differ amongst those who drop out and keep up with exercise. Thus, there is an association between non-conscious processes and whether people engage in physical activity. The targeting of implicit processes therefore appears to be a fruitful research avenue.

## Priming

One way that preconscious cognitive states have been successfully influenced is through priming which is the activation of mental processes through environmental stimuli (Bargh and Chartrand, 2000). This can be achieved at both the subliminal and supraliminal level. Supraliminal priming involves conscious awareness of the stimuli but not the resulting behavior. Subliminal, however, refers to the priming of processes beyond perception. Thus, one is unaware of both the stimulus and behavior.

Priming has been used extensively to target health-related behaviors relating to diet and healthy eating (e.g., Papies and Hamstra, 2010; Gaillet et al., 2013; Papies et al., 2014). For example, Papies et al. (2014) successfully reduced the purchasing of snacking items within overweight individuals. Concerning physical activity, however, priming research has been limited to date (Rebar et al., 2016). Indeed, the promotion of physical activity has relied heavily on the investigation of conscious, reflective processes.

## Reflective Processes

The use of priming is particularly concerned with non-conscious processes; that of which the individual is unaware. Research to promote physical activity, however, has largely focussed on conscious, deliberate processes (Sheeran et al., 2013). Popular theories within health psychology attempt to highlight the reflective processes influencing behavior to then target for intervention. Such processes include outcome expectancies, risk perceptions, normative factors, self-efficacy, and intentional states (Rosenstock, 1974; Ajzen, 1991; Bandura, 1991). For example, the Theory of Planned Behavior (TPB; Ajzen, 1991) suggests attitude, subjective norms and perceived behavioral control govern the development of a behavioral intention. This intention then proceeds to behavioral performance, so long as the behavior is under volitional control.

Although such processes are not necessarily expected to be rational, crucially they are theorized to undergo deliberation. For example, a choice made under pressure or stress could lead to the decision to not participate in physical activity; however, despite this irrationality, such outcomes are nonetheless consciously deliberated.

Despite the fact these models have accounted for significant variance in intention, the predictive validity in behavior is rather modest (Armitage and Conner, 2001; Webb and Sheeran, 2006). For example, Rhodes and de Bruijn (2013) found the intention-behavior gap to be 48% pertaining to physical activity. Furthermore, interventions altering conscious beliefs using the popular method of persuasive appeals have demonstrated limited success to date. Thus, engendering to change intentions does not necessarily result in actual behavior change (Rhodes and Dickau, 2012).

## Theoretical Integration

As scientific progress may be facilitated by the integration of behavior change theories (Hagger, 2009), hybrid models have been developed to facilitate the progression of behavior change. Due to the intention-behavior gap, these models have tended to focus on the links between intention formation (motivation) and intention enactment (volition). For example, the Rubicon Model of Action Phases (Heckhausen and Gollwitzer, 1987) states the importance of deliberative and implemental mind-sets. When an individual crosses the Rubicon (a metaphor for intentional commitment), the committed self terminates deliberation and proceeds to action through the initiation of plans. Similarly, the Health Action Process Approach (Schwarzer, 1992) integrates common reflective processes with planning strategies and various volitional self-efficacy beliefs. For example, upon the development of an intention (motivational phase), physical activity will only follow if the individual has the relevant efficacy beliefs and utilizes different plans (volitional phase). Thus, theory has identified two phases of change, with the implemental phase receiving more recent attention due to issues surrounding self-regulation.

In addition to focusing on the ‘right hand side’ of behavior change (i.e., behavior implementation), theories have also sought to address the motivational aspects of change. These aspects focus on the content of motivation and thus differ from the number of studies attempting to add explanatory value of intention and behavior through the addition of predictors.

Within the recently developed Integrated Behavior Change Model, Hagger and Chatzisarantis (2014) suggest that the Self Determination Theory (SDT; Deci and Ryan, 1985) can be integrated within the TPB. SDT is a macro theory of human behavior consisting of different subcomponents, of which includes a distinction between autonomous and controlled motivation. An autonomously motivated individual seeks pleasure and enjoyment in behavior whilst also having a sense of volition. Controlled motivation refers to externally influenced choices and perceived pressure to engage in behavior. It is assumed that those more intrinsically motivated will outperform those under controlled motivation. For example, individuals are more likely to participate in physical activity if their reasons concern self-satisfaction, enjoyment and pleasure. Conversely, individuals are less likely to engage in physical activity if they feel forced, pressurized and obligated to do so. The principle behind the model integration concerns the foundation of determinants specified within the TPB. More specifically, the theorized influences of intention are suggested to be underpinned by self-determined or controlled outcomes (Deci and Ryan, 2000). Thus, the ‘type’ of motivation within the SDT act as antecedents to the proximal predictors stated within the TPB (Hagger and Chatzisarantis, 2009). In addition to this integration, postintentional or volitional factors are accounted for within the model through the inclusion of the popular self-regulatory strategy of planning (Gollwitzer, 1999).

## Priming Social Cognitive Processes

As previously mentioned, priming has been used to directly change behavior. Bargh (2006), however, called for the evolution of the second wave of priming research, with such research attempting to explain how motives can be manipulated in the same way as overt behavior. In answering this call, research has successfully altered attitudes towards physical activity (e.g., Bluemke et al., 2010; Berry, 2016). For example, Bluemke et al. (2010) found physically active students responded better to automatic evaluations after affective primes than those students who were inactive.

In addition to priming attitudes, studies have also primed the ‘type’ of motivation mentioned above within both the SDT and Integrated Behavior Change Model. Specifically, priming has been found to be a valid technique for influencing the non-conscious activation of implicit autonomous and controlled motives. These studies take a dual-task paradigm with priming occurring at the afferent stage and behavioral performance at the efferent stage. Levesque and Pelletier (2003) were the first to examine SDT variables at the non-conscious level. The study involved integrating autonomy and controlled words (or heteronomous as mentioned in the study) within a Scrambled Sentence Test method (Srull and Wyer, 1979). It was found that those primed with autonomous words increased motivation compared to those primed with words related to control. Interestingly, priming affects have also replicated the results at the conscious level with those primed autonomously outperforming those subjected to a controlled prime (e.g., Pelletier et al., 2001; Radel et al., 2009; Banting et al., 2011). For example, Banting et al. (2011) found autonomously primed participants showed more positive behavioral tendencies, including prolonged cycling exercise.

In summary, not only can implicit motives be primed outside of conscious awareness, the content of motivation can also be manipulated. This manipulation can also mirror results found at the level of consciousness. As such, the non-conscious activation of mental representations offers potential avenues to target.

## Future Recommendations

Priming the content of motivation is a recent, yet innovative approach to changing physical activity that offers potential developments in the area. In addition to the content of motivation at the reflective level, other conscious processes may also be influenced implicitly. Included within several models of behavior change, one of the most popular reflective processes is self-efficacy (Bandura, 1989). Although self-efficacy is preceded with the word ‘perceived’ thus implying conscious deliberation, it could also be that repetition, experience and prior learning leads to the formation of non-conscious schemata. Thus, efficacy beliefs could also be influenced implicitly, with the activation of such processes playing an influential role in governing behavior. With a distinction made between action and coping self-efficacy (Schwarzer and Renner, 2000), it could be that these implicit efficacy beliefs operate differently at the preintention and postintention phases.

Furthermore, there could also be interactions between implicit processes occurring at the non-conscious level. Dual process models have highlighted individual differences with intention enactment. For example, a depleted state (Baumeister et al., 2007), lack of motivation and opportunity (Fazio, 1990), and executive function (Hall and Fong, 2007) have been suggested to dictate the role of implicit processes. Implicit processes, therefore, have been seen to travel in a linear direction. However, the interaction between implicit efficacy beliefs and implicit attitudes may be unidirectional. In line with the conscious level (Bandura, 1991), implicit efficacy beliefs may influence implicit attitudes. Furthermore, Williams and Rhodes (2016) have recently suggested that there is a two-way relation between motivation and self-efficacy at the conscious level. That is, not only does self-efficacy impact upon motivation (i.e., outcome expectancies), motivation may also be a cause of self-efficacy. Concerning the implicit level, it could also be that implicit motivation (i.e., attitudes) governs implicit self-efficacy. For example, a negative implicit attitude toward physical activity may lead to a decrease in non-conscious self-efficacy. Thus, the relationship between these non-conscious processes becomes reciprocal. Such work may be useful in increasing the predictive validity of behavior and highlighting potential avenues for interventions.

## Conclusion

In summary, research has addressed issues of physical activity change at the conscious level through the manipulation of reflective processes. Recently, however, non-conscious processes have been successfully influenced through behavioral priming. More specifically, priming has been demonstrated to successfully influence the content of motivation through the manipulation of both autonomous and controlled implicit processes. Future research could therefore attempt to implicitly alter other common reflective processes, such as self-efficacy, as well as their interactions at the non-conscious level. Such work could well lead to an increase in physical activity, particularly due to inconsistent intervention results at the conscious level.

## Author Contributions

The author confirms being the sole contributor of this work and approved it for publication.

## Conflict of Interest Statement

The author declares that the research was conducted in the absence of any commercial or financial relationships that could be construed as a potential conflict of interest.
